# Researchers’ perspectives on donor-level metadata collection and reporting for human tissue research

**DOI:** 10.1093/jamiaopen/ooag213

**Published:** 2026-11-06

**Authors:** Rachel Liu-Galvin, Kathy Tran, Mishal Khan, Michael T Eadon, Pinaki Sarder, Yulia A Levites Strekalova

**Affiliations:** Department of Health Services Research, Management and Policy, College of Public Health and Health Professions, University of Florida, Gainesville, FL 32610, United States; Department of Economics, College of Liberal Arts and Sciences, University of Florida, Gainesville, FL 32610, United States; Department of Health Services Research, Management and Policy, College of Public Health and Health Professions, University of Florida, Gainesville, FL 32610, United States; Divisions of Nephrology and Clinical Pharmacology, Indiana University School of Medicine, IN 46202, United States; Department of Medicine (Quantitative Health), College of Medicine, University of Florida, Gainesville, FL 32610, United States; Department of Health Services Research, Management and Policy, College of Public Health and Health Professions, University of Florida, Gainesville, FL 32610, United States

**Keywords:** tissue donors, biomedical research, reproducibility of results, confidentiality, ethics, research

## Abstract

**Introduction:**

No widely adopted guidelines exist for collecting and reporting donor-level metadata in tissue-based research, which limits interpretability and reproducibility and may introduce bias. This study aimed to identify researchers’ perspectives to inform ethical and appropriate metadata practices.

**Materials and Methods:**

Semi-structured interviews were conducted with 16 investigators from the Human BioMolecular Atlas Program. Thematic analysis using inductively derived codes identified metadata elements and perspectives on their collection and reporting.

**Results:**

Participants identified 80 metadata variables across six domains: demographic, sociodemographic, medical history, personally identifying information, cause of death, and tissue/organ data. Participants predominantly supported collection and reporting of demographics and medical history, whereas views on cause of death and sociodemographic data were mixed.

**Conclusion:**

These findings can inform the future development of ethical guidelines for donor-level metadata collection and reporting in tissue-based research. Future stakeholder engagement and consensus-building are needed to determine how individual metadata elements should be defined, collected, operationalized, and reported.

## Introduction

Human tissue research relies on donor samples, yet there remains no consensus on which donor information should be ethically and scientifically reported.^1^^,^^2^ While tissue processing protocols are described in detail, donor-level metadata such as age, sex, medical history, and socioeconomic indicators are inconsistently captured.^3^ These metadata provide important context for interpreting cellular and molecular findings by enabling researchers to examine relationships between donor phenotypic characteristics, such as clinical and socioeconomic history, with the cellular and molecular phenotypes generated from their biospecimens. These metadata may also provide important information about exposures affecting epigenetic regulation.^4^ Furthermore, this gap between human characteristics and cell-level findings creates a broken link in the data lifecycle, limiting reproducibility, introducing potential biases, and reducing the interpretability of results.^3^^,^^5^ These challenges underscore the need for standardized guidelines for reporting donor-level metadata in studies using human tissue samples.

While cell-level methods such as single-cell RNA sequencing, spatial transcriptomics, and spatial epigenomics have significantly advanced our understanding of human biology,^6–8^ less attention has been given to capturing human characteristics that provide important context for interpreting cellular and molecular phenotypes. Sociodemographic factors and medical and drug histories may reflect environmental exposures that influence biological processes, including through epigenetic mechanisms.^4^ These mechanisms underscore the importance of collecting donor-level metadata in tissue-based research, while balancing scientific value with privacy and identifiability concerns.^2^

Biospecimen studies often underrepresent minority groups and healthy controls, compromising external validity.^6^ Donor-level metadata are therefore crucial for characterizing the composition and representation of populations included in biospecimen collections and assessing the generalizability of findings. However, diversity is multidimensional, and the relevance of particular donor characteristics depends on the scientific question and intended inference.^7^ Biases in data and the variables used to develop predictive models may perpetuate disparities and lead to systematically biased predictions.^8^ Individual- and population-level descriptors such as race and ethnicity also require careful interpretation because they are socially constructed and should not be used as proxies for biological or genetic variation.^9^ More broadly, incomplete metadata can hinder reproducibility and the interpretability of findings from research using tissue-based datasets.^10^ The availability of linked clinical data is also an important consideration for researchers using human tissue samples.^11^ Promoting inclusive and transparent reporting is therefore critical. Despite this, to our knowledge, there are no widely adopted guidelines that define the specific demographic, clinical, and other contextual variables that constitute a complete donor profile for tissue-based research, specify the necessary granularity for each variable, and outline how to ensure consistent reporting. While guidelines exist for randomized trials,^12^ these are not specifically designed to address donor-level metadata in cell-level tissue research.

Furthermore, mapping participant-informed metadata to existing data models exposes structural limitations in how legacy systems handle complex social constructs. The 2025 NASEM guidelines^9^ emphasize separating self-identified race and ethnicity from genetic ancestry. However, many established clinical data models were originally designed around administrative billing or legacy clinical workflows that frequently conflate these domains or lack the structural flexibility to cleanly separate social experience from biological proxies. Therefore, adopting standards like Observational Medical Outcomes Partnership (OMOP) or Fast Healthcare Interoperability Resources (FHIR) for tissue metadata is not merely a plug-and-play mapping exercise. It requires actively profiling and constraining these models (e.g., through specific FHIR Implementation Guides) to ensure they structurally enforce the separation of social constructs from biological variables.

The objective of this exploratory study was to identify donor-level metadata categories and elements considered important by researchers and to characterize their perspectives on which metadata should be collected and reported. The study was not designed to establish how individual metadata elements should be defined, measured, operationalized, or implemented. To our knowledge, this is the first study to systematically examine research obtaining researchers’ perspectives on the appropriate and ethical collection and reporting of donor-level metadata in tissue-based research. Using interviews with investigators from the National Institutes of Health (NIH)-funded Human BioMolecular Atlas Program (HuBMAP), we identified areas of consensus and disagreement on which donor-level metadata should be collected or reported, providing a participant-informed baseline from which to initiate a broader community effort to standardize the human side of tissue-based research.

## Methods

### Setting and participants

This qualitative elicitation study recruited participants from the NIH-funded HuBMAP consortium, an international initiative focused on mapping the human body at cellular resolution.^13^ HuBMAP investigators work with human tissue samples and associated data, making them well-positioned to provide insight into which donor-level metadata should be collected for tissue-based research.

Participants were purposively sampled from active HuBMAP members in leadership or senior technical roles (e.g., principal investigators, professors, research scientists, and laboratory directors) to capture perspectives across key workstreams, including tissue procurement, experimental biology, data generation, and bioinformatics. Participants represented nine institutions across academia, healthcare, and biotechnology sectors, providing diverse perspectives on human tissue donation.

Participants were contacted via email up to three times. Recruitment was guided by information power considerations to ensure sufficient depth in gathering researchers’ perspectives to inform future donor-level metadata collection and reporting recommendations.^14^

### Interview data collection

Fifteen interviews were conducted with sixteen participants (including one involving two participants) via secure video conferencing between July and November 2024. Participants received a standardized explanation of the project and provided verbal consent prior to participation. Interviews were conducted individually by trained qualitative researchers with no prior relationship to participants to minimize social desirability bias. A semi-structured guide (see Supplementary Appendix A) explored perspectives on representation in research, donor tissue heterogeneity, and implications for data quality and scientific advancement. Interviews lasted between 6 and 31 min (mean: 17 min) and were audio-recorded and transcribed verbatim using Microsoft 365 Transcribe, with manual verification for accuracy.

### Data analysis

Transcripts were cleaned (e.g., spelling, abbreviations, and filler words) prior to analysis. The lead author developed a preliminary codebook through iterative review of the transcripts, which was refined in collaboration with coauthors. Thematic analysis was conducted using inductively derived codes to capture the full breadth of metadata elements mentioned by participants. The final codebook (see Supplementary Appendix B) categorized variables into demographic information, sociodemographic information, medical history, personally identifying information, cause of death, and tissue or organ data (e.g., sample quality, viability, and other sample features). For each variable, participants’ views were classified into three categories: (1) collect and report, (2) collect but not report, or (3) neither collect nor report. A presence/absence-based coding approach was used, with each variable coded once per interview to prioritize breadth of perspectives.

Variables were classified as “collect and report” unless participants explicitly recommended otherwise; routine practices were interpreted as endorsements unless objections were noted. This interpretative approach was considered appropriate given the open-ended nature of the interview questions and is consistent with established qualitative research practices, in which inference and interpretation are integral to the analytic process.^15^

Two investigators independently coded all transcripts, followed by a joint reconciliation phase to achieve consensus. Analyses were conducted using R version 4.4.1 and RStudio.

### Ethical review

This study was reviewed and approved as exempt by the Institutional Review Board (IRB) at the University of Florida ET00041509.

## Results

We conducted 15 interviews with 16 participants (including one two-participant interview) and asked them for their opinions on the data that should be collected from tissue donors. Our thematic analysis identified 80 donor-level metadata variables, consolidated into 25 variable types. Variable types were further grouped into six categories: (1) medical history, (2) demographic information, (3) sociodemographic information, (4) cause of death, (5) tissue or organ data (e.g., sample quality, viability, or other sample features), and (6) personally identifying information. Participants recommended whether each variable be (1) collected and reported, (2) collected but not reported, or (3) neither collected nor reported. Supplementary Appendix C provides full variable mapping by transcript; aggregate frequencies are summarized in Supplementary Appendix D. Consolidated category frequencies, stratified by recommendation type, are presented in Table 1, which lists variable categories in order of “collect and report” recommendations. Supplementary Appendix Fig. SE shows the distribution of recommendations by variable type.

**Table 1.** Frequency of collection and reporting recommendations from HuBMAP investigators.

Category	Variable type	Recommendation		
		Collect and report	Collect only	Do not collect
**Medical history**	Physiological measurements	15	1	0
	Laboratory test results	12	3	0
	Diagnosed medical conditions	11	0	0
	Medical history (general)	10	0	0
	Substance use	6	0	0
	Medication history	5	0	0
	Dietary history	4	0	0
	Pregnancy	4	0	0
	Embryo/fetus/infant status	3	0	0
	Pediatric medical history	3	0	0
**Demographic information**	Race	11	0	0
	Age	10	0	0
	Ethnicity	9	0	0
	Sex	8	0	0
	Gender	6	0	0
	Age (publish only with the consent of donor’s family/legal representative)	1	0	0
	Demographic information (general or unspecified)	1	0	0
	Race (publish only with the consent of donor’s family/legal representative)	1	0	0
	Race (participant emphasized race should only be reported with donor consent)	0	1	0
**Sociodemographic information**	Sociodemographic context	8	3	2
**Cause of death**	Cause of death	3	1	0
	Mechanism of death	2	1	0
**Tissue or organ data**		2	0	0
	Sample features—anatomical characteristics	2		
	Sample features—molecular data	1	3	0
	Sample quality	3	0	0
**Personally identifying information**	Personally identifying information	1	4	12

Most variables within the medical history category were recommended for full collection and reporting. Physiological measurements (e.g., Body Mass Index (BMI)) received the most mentions, followed by laboratory test results; diagnosed conditions and general medical histories were also frequently cited. However, a few participants suggested withholding certain measurements or test results from publications. Some participants also suggested collecting lifestyle data, such as diet, physical activity, and substance use beyond tobacco and alcohol.

Demographics were overwhelmingly recommended for collection and reporting. Of these, race and age were most frequently mentioned, followed by ethnicity, sex, and gender. A minority noted some demographics, particularly race, should only be published with donor consent. Participants discussed race and ethnicity in relation to donor representativeness, while also recognizing limitations in their interpretation. One participant explicitly distinguished biological ancestry from race, describing the latter in relation to identity and lived social experience. The participant characterized race as a “man-made construct” and suggested that area-level socioeconomic measures derived from ZIP code may provide additional information about social context.

Views on sociodemographic context were mixed, with collection and reporting recommended by most participants, while some suggested this information, particularly socioeconomic status and geographic location, should be collected but not reported, or omitted altogether. Several participants recommended collecting socioeconomic status. One participant described the potential value of using ZIP codes to derive area-level socioeconomic disadvantage, explaining that this information could help tease out molecular or health factors associated with contextual exposures, such as living in a “nutritional food desert.” However, another participant distinguished between the value of retaining geographic information internally and the risks of public reporting, noting that ZIP code could be used to derive social determinants of health and environmental exposures but that “certainly we can’t release that publicly, especially because we have a small sample size.”

For tissue samples obtained from deceased donors, few participants recommended recording the cause and mechanism of death. Among those who did, most recommended collecting and reporting it, while a minority recommended collection only, omitting its inclusion in publications.

Participants also identified characteristics of tissue or organ samples, including anatomical site, sample quality, and molecular features, as potentially important metadata. Because the aim of this study was to identify donor-level metadata considered important by researchers, these specimen-level variables were captured during coding and reported in Table 1 but were not incorporated into the participant-informed recommendations summarized in Table 2, which focus on donor-level metadata. Personally identifying information was discussed frequently, with most participants recommending that this data be neither collected nor reported. A few participants suggested certain information, e.g., ZIP code and Social Security Number, be collected but not reported. Thus, ZIP code illustrated a point of tension between the potential scientific value of sociodemographic information and concerns regarding donor privacy and re-identification. Based on our findings, we summarized participants’ perspectives on the collection and reporting of donor-level metadata by level of agreement. Areas with strong support may represent candidate priorities for future consensus-building, whereas areas with mixed perspectives highlight issues requiring further investigation and stakeholder engagement. These findings are summarized in Table 2.

**Table 2.** Summary of participant perspectives on donor-level metadata collection and reporting.

**Level of agreement**	**Metadata elements**	**Summary of participant perspectives**
**Strong support for collection and reporting**	Demographic information: age, race, ethnicity, sex, and gender	Frequently recommended for collection and reporting; a minority recommended reporting age or race only with donor or family consent
	Medical history: physiological measurements (e.g., height, weight, BMI, laboratory results, and blood pressure), laboratory test results, diagnosed medical conditions, substance use, medication history, dietary history, pregnancy-related medical history (if applicable), medical history related to the embryo, fetus, or infant (if applicable), and pediatric medical history (if applicable)	Generally recommended for collection and reporting, although some participants recommended collecting but not reporting particular physiological measurements or laboratory results
**Mixed perspectives**	Cause and mechanism of death	Participants who discussed these elements generally supported collection, but differed regarding whether they should be reported, with concerns regarding privacy and re-identification
	Sociodemographic context	Participants differed regarding whether sociodemographic information should be collected and reported, collected but not reported, or not collected, with concerns regarding privacy and re-identification
**Strong support against collection**	Personally identifying information, e.g., name, address, zip code, telephone numbers, and social security number	Most participants opposed collection because of privacy and re-identification concerns, although some participants supported collecting particular elements without reporting them

## Discussion

### Summary of the present study

This study provides a novel, stakeholder-informed perspective on the importance of capturing donor-level metadata in tissue-based research. Since data use and ethical requirements vary across institutions and jurisdictions, this study does not seek to establish a metadata standard, but rather to document investigators’ views to inform future guidelines. This exploratory study identified broad donor-level metadata categories and elements considered important by researchers, but it does not establish how individual elements should be defined, measured, operationalized, or implemented. For example, substance use was identified as an important metadata element, but it was not ascertained whether smoking should be characterized by smoking status, frequency, duration, or cumulative exposure. Obtaining and standardizing lifestyle information may also present practical challenges in tissue-donation settings, as such information may depend on medical records or next-of-kin reports or require instruments such as dietary recall questionnaires or wearable devices. Similarly, identifying physiological measurements as an important metadata element does not establish which specific anthropometric measurements should be collected or how they should be interpreted across populations. Several participants recommended collecting socioeconomic status, an important non-clinical determinant of health.^4^ Area-level measures such as the Area Deprivation Index (ADI)^16^ may provide one approach to operationalizing socioeconomic context, although reliance on ZIP code raises privacy and re-identification concerns. Further research and consensus-building are therefore needed to determine the appropriate granularity, operational definitions, and methods of collection for individual metadata elements.

Participants’ recommendations regarding race and ethnicity require careful interpretation in light of evolving guidance on their use in research.^9^ Although participants frequently recommended their collection and reporting, this finding reflects participants’ perspectives and should not be interpreted as endorsing routine reporting or their use as proxies for biological or genetic variation. Notably, one participant explicitly distinguished ancestry from race, describing differences between biological ancestry, identity, and lived social experience. This distinction is consistent with the 2025 National Academies recommendations that researchers avoid using race as a proxy for biological or genetic variation and clearly define the concepts being measured.^9^ Future development of donor-level metadata standards should therefore distinguish among race, ethnicity, genetic ancestry, and relevant social or environmental measures, specify the scientific purpose for collecting each construct, and determine appropriate methods for their collection and reporting.

Most participants expressed strong support for collecting and reporting demographic information and medical history, whereas cause and mechanism of death and sociodemographic context elicited mixed views. These findings provide an initial evidence base to inform the development of guidelines for collecting and reporting donor-level metadata in tissue-based research. An important next step in translating these recommendations into practice is to consider how the identified metadata elements could be represented interoperably across biobanks, repositories, information systems, and research platforms. This enables data to be shared, integrated, reused, and reproduced.

To illustrate how the participant-informed recommendations identified in this study could align with existing biomedical informatics frameworks, we developed Supplementary Appendix Table SF, which provides an illustrative mapping of the recommended donor-level metadata elements to widely used biomedical informatics data models (e.g., FHIR,^17^ OMOP CDM,^18^ and MIABIS^19^) and standardized terminologies (e.g., SNOMED CT,^20^ LOINC,^21^ and RxNorm^22^). This mapping demonstrates how the metadata elements identified in this study could be represented within existing biomedical informatics frameworks to support future interoperability efforts. It is illustrative rather than prescriptive and does not constitute a validated implementation specification or proposed metadata standard. While the mapping of data elements presented in Supplementary Appendix Table SF suggests potential alignment with models such as FHIR and OMOP CDM, translating broad stakeholder recommendations into these rigid ontologies reveals significant semantic gaps. Participants in our study frequently endorsed high-level categories, such as “substance use” or “socioeconomic status,” yet standardized terminologies like SNOMED CT and LOINC require highly granular, operationalized concepts (e.g., distinguishing between a LOINC code for ‘current smoking status’ versus ‘pack-years’). This granularity gap highlights that qualitative consensus on a variable’s importance does not translate seamlessly into a single ontological code. Future standards development must bridge this divide by determining the minimum viable granularity required for tissue research without overburdening data collectors. Finally, a notable structural gap exists regarding data provenance. Established ontologies like OMOP CDM are primarily optimized to capture data from direct clinical encounters with living patients via Electronic Health Records (EHRs). In human tissue research, however, metadata is frequently acquired postmortem via surrogate reporting, next-of-kin interviews, or fragmented medical records. Translating these donor-level elements into standard ontologies will require robust extensions to capture the provenance, source, and reliability of the data, ensuring that downstream researchers understand whether a phenotypic trait was clinically measured, self-reported by the donor, or estimated by a surrogate.

### Strengths and limitations

A key strength of this study was the integration of insights from investigators engaged in tissue-based research, supporting the development of future stakeholder-informed guidelines that balance scientific utility with participant privacy and practical feasibility. To our knowledge, this is the first study to systematically explore and identify participant-informed recommendations for donor-level metadata in tissue-based research. Existing reporting standards such as CONSORT, STROBE, or the National Cancer Institute (NCI)’s Best Practices for Biospecimen Resources address clinical, trials, epidemiological studies, and oncology, respectively, but do not specify which donor-level metadata should be published in tissue-based studies.^23–25^ This leaves a clear gap that our findings aim to address. Our recommendations align with the NCI’s minimal clinical data set for annotating biospecimen resources, with several variables in common, including demographics, medical history, and lifestyle factors. While several specimen-level variables were also mentioned by participants, they were not included in our final table of recommendations because our aim was to identify donor-level metadata to contextualize cell-level findings.

Several limitations of this study should be noted. First, high participant agreement occurred primarily at the level of broad metadata categories, such as laboratory blood tests or substance use, which does not imply consensus on specific variable definitions, granularity, or operationalization, particularly for complex constructs like race, ethnicity, and genetic ancestry. Furthermore, race, ethnicity, and ancestry were not systematically defined during the interviews, nor were participants systematically asked whether or how these constructs should be collected. Thus, participants’ recommendations regarding these metadata elements should be interpreted as perspectives on the perceived importance of these elements rather than consensus regarding their definitions, measurement, or appropriate use. Relatedly, the metadata crosswalk to existing data models (e.g., FHIR, OMOP CDM) remains conceptual and has not been formally validated. Second, detailed participant demographics and institutional affiliations were not recorded, preventing subgroup analyses and triangulation. Finally, while independent coding minimized bias, coders who did not conduct interviews may have missed contextual nuances inherent in semi-structured interview data.

### Implications for future research

Building on these initial findings, future research should move from broad recommendations toward defining and operationalizing granular metadata specifications, particularly for demographic and genomic constructs. Formal pilot testing and informatics validation are also needed to evaluate the real-world utility and technical feasibility of the proposed data-model crosswalk. Additionally, future work should capture detailed participant characteristics to examine how metadata priorities vary across professional roles, research domains, and institutional types. Finally, combining qualitative methods with quantitative consensus frameworks (e.g., Delphi studies) or observational implementation trials could help validate these findings and minimize interpretive subjectivity.

Together, the findings and the illustrative crosswalk presented in this paper provide a foundation for subsequent consensus-building and operationalization of donor-level metadata, including the development of standardized definitions and measurement approaches. Future studies should gather input from a wider variety of stakeholders, including end users of tissue-based research data, data scientists responsible for organizing and managing these data, potential donors and donor advocacy groups, tissue and organ procurement organizations, transplant organizations such as the United Network for Organ Sharing (UNOS), and regulatory and standards organizations, to capture diverse perspectives. Incorporating these perspectives will be important for determining what metadata can and should be collected, how it should be represented and managed, how scientific utility should be balanced with donor preferences and privacy, as well as practical feasibility, and how donor metadata should be obtained when self-report is not possible, as may be the case for deceased donors. Areas that elicited mixed views warrant further clarification through consensus-building approaches such as Delphi expert panels. Developing and adopting clear, ethical donor metadata guidelines is essential for advancing accurate, equitable, reproducible, and generalizable tissue-based research that translates into improved population health.

## Supplementary Material

ooag213_Supplementary_Data

## Data Availability

The data for this study are available from the authors upon reasonable request.
