# Supplementary material for: Researchers’ perspectives on donor-level metadata collection and reporting for human tissue research
Source: JAMIA Open. 2026 Nov 6;9(6):ooag213. doi: 10.1093/jamiaopen/ooag213 (PMC13743425; doi:10.1093/jamiaopen/ooag213)
Supplement: Supplementary file 1 [file ooag213_supplementary_data.zip › JAMIA Appendix A.docx]

**Appendix A. Interview Questions.**

1. Can you please briefly describe your current academic position and role within the HuBMAP project?
a. What types of tissues do you work with?

2. In your opinion, what data about tissue donors should be collected. Please list all data elements that come to mind.
a. What else?
b. Any other elements?

3. What information about tissue donors should be retained and shared as meta-data for each sample? Again, please list all data elements that you think should be included.
a. Are there any data elements that should be collected but not saved as meta-data for each tissue sample?

4. What information about tissue donors should be included in the publications?
a. What elements should be collected but not required for reporting and why?

5. If not addressed earlier. **SKIP IF RACE AND ETHNCITY HAVE BEEN MENTIONED in QQ2, 3, or 4.** In your opinion, what demographic data like race, or socio-economic status should be collected for tissue donors and why?
a. Should these data be stored as meta-data for all tissue samples?
b. Should these data be included in the academic papers that report on cell- and tissue-level research?
c. What ethical considerations and challenges do you foresee with reporting the demographics of tissue donors in cell-level research?

6. What is your current experience and practice in obtaining and reporting tissue donor characteristics in your research?
a. How do you use those data?
b. What challenges, if any, do you experience?
