# Supplementary material for: Researchers’ perspectives on donor-level metadata collection and reporting for human tissue research
Source: JAMIA Open. 2026 Nov 6;9(6):ooag213. doi: 10.1093/jamiaopen/ooag213 (PMC13743425; doi:10.1093/jamiaopen/ooag213)
Supplement: Supplementary file 1 [file ooag213_supplementary_data.zip › JAMIA Appendix B.docx]

**Appendix B. Codebook for Analysis of Interview Transcripts.**

| **Codes**  **(Broad metadata categories)** | **Definition** | **When to Use This Code** | **Example Quotes** | **Subcodes**  **(Metadata elements within each category)** | **Collect and Report** (Indicate whether the researcher stated that this information should be both collected and reported.) | **Collect Only (Do Not Report)** (Indicate whether the researcher stated that this information should be collected but not reported.) | **Do Not Collect or Report** (Indicate whether the researcher stated that this information should not be collected or reported.) |
| --- | --- | --- | --- | --- | --- | --- | --- |
| **Demographic Information** | Basic personal data about the donor | When the researcher states that demographic information about the donor should or should not be collected or reported | “We currently have age, race, ethnicity, gender and sex. Those would be sort of your minimum criteria” | ·       Age |  |  |  |
|  |  |  |  | ·       Sex |  |  |  |
|  |  |  |  | ·       Gender |  |  |  |
|  |  |  |  | ·       Race |  |  |  |
|  |  |  |  | ·       Ethnicity |  |  |  |
|  |  |  |  | ·      Other (specify) |  |  |  |
| **Sociodemographic Information** | Social and economic characteristics | When the researcher states that sociodemographic information about the donor should or should not be collected or reported | That would be really important because I don't think that we've really teased out people who are in a geographical region. You can usually correlate by zip code that is socioeconomically on average disadvantaged. Sometimes you can correlate that with large data sets. Then that can help to tease out kind of molecular or health factors that are as a result of being in, for example, a nutritional food desert. Right now we don't record that information, but in an ideal world going forward, we would include that and make it available. | ·       Socioeconomic status/ socioeconomic disadvantage score |  |  |  |
|  |  |  |  | ·       Education level |  |  |  |
|  |  |  |  | ·       Health insurance |  |  |  |
|  |  |  |  | ·       Other (specify) |  |  |  |
| **Medical History** | Past illnesses and health conditions of the donor | When the researcher mentions that information about the donor’s health or medical history should or should not be collected or reported | “The ideal situation for us is that we would get a redacted medical record from our donors” | ·       Physiological measurements e.g. BMI, blood pressure, oxygen saturation |  |  |  |
|  |  |  |  | ·       Laboratory test values e.g., HbA1C, estimated glomerular filtration rate (eGFR), blood type, antibodies |  |  |  |
|  |  |  |  | ·      Diagnosed medical conditions/chronic illnesses/comorbidities e.g., hypertension, chronic kidney disease |  |  |  |
|  |  |  |  | ·       Disease stage/severity |  |  |  |
|  |  |  |  | ·       Past infections |  |  |  |
|  |  |  |  | ·       Medication history |  |  |  |
|  |  |  |  | ·       Substance use e.g., smoking, alcohol, recreational drugs |  |  |  |
|  |  |  |  | ·       Dietary history |  |  |  |
|  |  |  |  | ·       Previous surgeries |  |  |  |
|  |  |  |  | ·       History of ventilation or breathing assistance |  |  |  |
|  |  |  |  | ·       Whether born preterm (for pediatric samples) |  |  |  |
|  |  |  |  | ·       Congenital conditions/Birth defects e.g., hypoplastic kidneys (for pediatric samples) |  |  |  |
|  |  |  |  | ·       Pregnancy-related information e.g. gestation period, maternal age/BMI, fetal sex, complications e.g., preeclampsia or intra-uterine growth restriction (IUGR), APGAR score of newborn (for placenta samples) |  |  |  |
|  |  |  |  | ·       Genetic conditions |  |  |  |
|  |  |  |  | ·       Epigenetic data or RNA sequence data |  |  |  |
|  |  |  |  | ·      Other (specify) |  |  |  |
| **Personally Identifying Information** | Data that can directly identify a donor | When the researcher discusses personally identifying information that should or should not be collected/reported. | “We would omit any personal identifying information” | ·       Name |  |  |  |
|  |  |  |  | ·       Address |  |  |  |
|  |  |  |  | ·       Zip code |  |  |  |
|  |  |  |  | ·       Telephone number |  |  |  |
|  |  |  |  | ·       Cause of death (if identifying) |  |  |  |
|  |  |  |  | ·       Family member information |  |  |  |
| **Cause of Death** | The specific reason or condition leading to the donor’s death | When the researcher discusses whether cause of death should be collected/reported. | “What other stuff would we not share? […] maybe how someone died” | ·       Disease-related cause |  |  |  |
|  |  |  |  | ·       Trauma-related cause |  |  |  |
|  |  |  |  | ·       Brain death vs. cardiac death |  |  |  |
|  |  |  |  | ·      Other (specify) |  |  |  |
| **Tissue or Organ Sample Quality and Viability** | Information on factors affecting the usability and condition of the donated tissue sample | When the researcher discusses factors impacting tissue integrity and viability for use in research | “Cold ischemic time is the amount of time it's on ice before we process it, so we had to have a really strict cut off, no more than 12 hours ischemic time” | ·       Warm ischemic time |  |  |  |
|  |  |  |  | ·       Cold ischemic time |  |  |  |
|  |  |  |  | ·       Processing time |  |  |  |
|  |  |  |  | ·       Type of processing performed |  |  |  |
|  |  |  |  | ·      Other (specify) |  |  |  |
| **Tissue or Organ Sample Features** | Specific details related to the organ or sample | When the researcher discusses features of the sample that may impact its use in research | “If they remove the entire kidney, we get a non-tumorous part […] we’ve had to think about kind of proximity to the tumor and we make that information available just so that people know that when they use our data”. | ·       Histology/microscopy/cell-level features |  |  |  |
|  |  |  |  | ·       Genomic/sequencing data |  |  |  |
|  |  |  |  | ·       Imaging e.g., optomap of an eye |  |  |  |
|  |  |  |  | ·       Unusual substances/unknown species detected in the sample |  |  |  |
|  |  |  |  | ·       Proximity to tumor for non-tumorous samples removed from a cancerous organ |  |  |  |
|  |  |  |  | ·       Fitzpatrick scale (skin) |  |  |  |
|  |  |  |  | ·       Pathology-determined damage (e.g., UV damage to the skin) |  |  |  |
|  |  |  |  | ·     Anatomical region sample was taken from |  |  |  |
|  |  |  |  | ·      Other (specify) |  |  |  |
