# Supplementary material for: Researchers’ perspectives on donor-level metadata collection and reporting for human tissue research
Source: JAMIA Open. 2026 Nov 6;9(6):ooag213. doi: 10.1093/jamiaopen/ooag213 (PMC13743425; doi:10.1093/jamiaopen/ooag213)
Supplement: Supplementary file 1 [file ooag213_supplementary_data.zip › JAMIA Appendix C.docx]

**Appendix C. Recorded Codes, Subcodes, and Collection and Reporting Recommendations by Interview.**

| Interview ID | Code | Subcode/s | Collect & Report? | Collect Only? | Do Not Collect? |
| --- | --- | --- | --- | --- | --- |
| 1 | Medical History | Physiological measurements (BMI), Diagnosed medical conditions, Past infections | x |  |  |
| 1 | Personally Identifying Information | Name, HIPAA-protected information (any), UNOS ID |  |  | x |
| 1 | Demographic Information | Age, Sex, Ethnicity, Race | x |  |  |
| 1 | Sociodemographic Information | Location (broad geographic region of donor residence) |  |  | x |
| 1 | Personally Identifying Information | Socioeconomic disadvantage score (based on zip code) | x |  |  |
| 1 | Tissue or Organ Sample Quality and Viability | Cold ischemic time | x |  |  |
| 2 | Demographic Information | Age, Sex, Ethnicity, Gender | x |  |  |
| 2 | Sociodemographic Information | Socioeconomic status |  |  | x |
| 2 | Medical History | Substance use (smoking), Substance use (cannabis smoking), Dietary history, Diagnosed medical conditions, History of ventilation or breathing assistance, Laboratory test results (eGFR) | x |  |  |
| 2 | Tissue or Organ Sample Quality and Viability | Processing time | x |  |  |
| 2 | Tissue or Organ Sample Features | Unusual substances/unknown species detected in the sample | x |  |  |
| 2 | Sociodemographic Information | Location (broad geographic region of donor residence) |  | x |  |
| 2 | Cause of Death | Cause of death, Mechanism of death | | x |  |
| 3 | Demographic Information | Age, Sex, Ethnicity, Race, Gender | x |  |  |
| 3 | Medical History | Physiological measurements (weight), Physiological measurements (height), Diagnosed medical conditions, Medication history | x |  |  |
| 3 | Sociodemographic Information | Location (broad geographic region of donor residence) | x |  |  |
| 4 | Medical History | Diagnosed medical conditions, Medication history | x |  |  |
| 4 | Demographic Information | Race, Ethnicity | x |  |  |
| 4 | Sociodemographic Information | Socioeconomic status |  | x |  |
| 4 | Personally Identifying Information | Zip code |  |  | x |
| 5 | Medical History | Laboratory test results (standard blood panel), Laboratory test results (standard metabolic panel), Diagnosed medical conditions, Medication history, Previous surgeries, Medical history (intraoperative details from organ recovery surgery e.g. blood pressure or ventilator settings), Laboratory test results (blood type), Laboratory test results (immunologic profiling), Laboratory test results (histocompatibility profiling) | x |  |  |
| 5 | Demographic Information | Demographic Information (general or unspecified) | x |  |  |
| 5 | Tissue or Organ Sample Quality and Viability | Warm ischemic time | x |  |  |
| 6 | Medical History | Medical history (general or unspecified) | x |  |  |
| 6 | Demographic Information | Age, Race, Ethnicity, Gender | x |  |  |
| 6 | Sociodemographic Information | Location (broad geographic region of donor residence), | | x |  |
| 6 | Personally Identifying Information | Personally identifying information (general or unspecified), Social Security Number, Zip code |  | x |  |
| 6 | Sociodemographic Information | Socioeconomic status | x |  |  |
| 7 | Demographic Information | Age, Race, Gender | x |  |  |
| 7 | Medical History | Substance use (smoking), Substance use (drug use), Substance use (alcohol), Physiological measurements (BMI), Diagnosed medical conditions, Dietary history, Laboratory test results (HbA1C), Laboratory test results (HLA) | x |  |  |
| 7 | Medical History | Laboratory test results (amylase), Laboratory test results (lipase), Laboratory test results (blood type) |  | x |  |
| 7 | Cause of Death | Cause of death, Mechanism of death | x |  |  |
| 8 | Demographic Information | Sex, Race, Ethnicity | x |  |  |
| 8 | Sociodemographic Information | Socioeconomic status, Location (broad geographic region of donor residence) | x |  |  |
| 8 | Tissue or Organ Sample Features | Molecular and genomic data (epigenetic data), Molecular and genomic data (RNA sequence data) |  | x |  |
| 8 | Medical History | Physiological measurements (oxygen saturations) |  | x |  |
| 8 | Medical History | Substance use (smoking), Diagnosed medical conditions, Pediatric medical history - history of genetic anomalies, Pediatric medical history - history of preterm birth, Pediatric medical history - hypoplastic organs | x |  |  |
| 9 | Demographic Information | Race, Sex, Age, Gender | x |  |  |
| 9 | Medical History | Physiological measurements (height), Physiological measurements (weight), Physiological measurements (BMI), Diagnosed medical conditions, Dietary history, Laboratory test results (metabolites or lipids) | x |  |  |
| 9 | Sociodemographic Information | Socioeconomic status | x |  |  |
| 10 | Medical History | Information about pregnancy (general or unspecified), Diagnosed medical conditions, Physiological measurements (BMI), Medication history | x |  |  |
| 10 | Demographic Information | Age, Race, Ethnicity | x |  |  |
| 10 | Personally Identifying Information | Personally identifying information (general or unspecified) | | | x |
| 11 | Medical History | Information about embryo/fetus/infant (fetal sex), Information about pregnancy (gestational duration), Information about pregnancy (gestational diabetes of mother), Information about embryo/fetus/infant (intra-uterine growth restriction (IUGR) of fetus), Information about pregnancy (preeclampsia of mother), Information about embryo/fetus/infant (APGAR of newborn), Physiological measurements (BMI), Physiological measurements (weight), Physiological measurements (height), Laboratory test results (blood type), Laboratory test results (general or unspecified), Medical history (general or unspecified) | x |  |  |
| 11 | Demographic Information | Age, Race, Ethnicity, Sex |  |  |  |
| 11 | Sociodemographic Information | Social history | x |  |  |
| 11 | Cause of Death | Cause of death, Mechanism of death | x |  |  |
| 11 | Personally Identifying Information | Personally identifying information (general or unspecified) | | | x |
| 12 | Personally Identifying Information | Address, Telephone number | |  | x |
| 12 | Demographic Information | Ethnicity, Sex, Age, Race | x |  |  |
| 12 | Sociodemographic Information | Socioeconomic status, Education level | x |  |  |
| 12 | Medical History | Medical history (general or unspecified), Medication history, Dietary history, Lifestyle, Family medical history | x |  |  |
| 12 | Personally Identifying Information | Other personally identifying information aside from address and telephone number |  | x |  |
| 13 | Demographic Information | Sex, Age, Race, Gender | x |  |  |
| 13 | Demographic Information | Race (participant emphasized race should only be reported with donor consent) | | x |  |
| 13 | Medical History | Diagnosed medical conditions, Laboratory test results (metabolites or lipids) | x |  |  |
| 14 | Personally Identifying Information | Personally identifying information (general or unspecified), Name, Address, Zip code |  |  | x |
| 14 | Demographic Information | Age, Sex, Ethnicity, Race | x |  |  |
| 14 | Medical History | Physiological measurements (BMI), Physiological measurements (height), Physiological measurements (weight), Diagnosed medical conditions | x |  |  |
| 14 | Cause of Death | Cause of death | x |  |  |
| 15 | Demographic Information | Age (publish only with consent of donor's family/legal representative), Race (publish only with consent of donor's family/legal representative) | x |  |  |
| 15 | Medical History | Medical history (publish only with consent of donor's family/legal representative), Physiological measurements (body measurements (not specified)) (publish only with consent of donor's family/legal representative) | x |  |  |
