# Supplementary material for: Researchers’ perspectives on donor-level metadata collection and reporting for human tissue research
Source: JAMIA Open. 2026 Nov 6;9(6):ooag213. doi: 10.1093/jamiaopen/ooag213 (PMC13743425; doi:10.1093/jamiaopen/ooag213)
Supplement: Supplementary file 1 [file ooag213_supplementary_data.zip › JAMIA Appendix D.docx]

**Appendix D. Frequency of All Codes and Subcodes with Collection and Reporting Recommendations from HubMAP Investigators.**

| **Code** | **Subcode** | **Collect and Report** | **Collect Only** | **Do Not Collect** |
| --- | --- | --- | --- | --- |
| Medical History | Diagnosed medical conditions | 11 | 0 | 0 |
| Medical History | Physiological measurements (BMI) | 6 | 0 | 0 |
| Medical History | Medication history | 5 | 0 | 0 |
| Medical History | Dietary history | 4 | 0 | 0 |
| Medical History | Physiological measurements (height) | 4 | 0 | 0 |
| Medical History | Physiological measurements (weight) | 4 | 0 | 0 |
| Medical History | Laboratory test results (blood type) | 2 | 1 | 0 |
| Medical History | Medical history (general or unspecified) | 3 | 0 | 0 |
| Medical History | Substance use (smoking) | 3 | 0 | 0 |
| Medical History | Laboratory test results (metabolites or lipids) | 2 | 0 | 0 |
| Medical History | Family medical history | 1 | 0 | 0 |
| Medical History | History of ventilation or breathing assistance | 1 | 0 | 0 |
| Medical History | Information about embryo/fetus/infant (APGAR of newborn) | 1 | 0 | 0 |
| Medical History | Information about embryo/fetus/infant (fetal sex) | 1 | 0 | 0 |
| Medical History | Information about embryo/fetus/infant (intra-uterine growth restriction (IUGR) of fetus) | 1 | 0 | 0 |
| Medical History | Information about pregnancy (general or unspecified) | 1 | 0 | 0 |
| Medical History | Information about pregnancy (gestational diabetes of mother) | 1 | 0 | 0 |
| Medical History | Information about pregnancy (gestational duration) | 1 | 0 | 0 |
| Medical History | Information about pregnancy (preeclampsia of mother) | 1 | 0 | 0 |
| Medical History | Laboratory test results (HLA) | 1 | 0 | 0 |
| Medical History | Laboratory test results (HbA1C) | 1 | 0 | 0 |
| Medical History | Laboratory test results (amylase) | 0 | 1 | 0 |
| Medical History | Laboratory test results (eGFR) | 1 | 0 | 0 |
| Medical History | Laboratory test results (general or unspecified) | 1 | 0 | 0 |
| Medical History | Laboratory test results (histocompatibility profiling) | 1 | 0 | 0 |
| Medical History | Laboratory test results (immunologic profiling) | 1 | 0 | 0 |
| Medical History | Laboratory test results (lipase) | 0 | 1 | 0 |
| Medical History | Laboratory test results (standard blood panel) | 1 | 0 | 0 |
| Medical History | Laboratory test results (standard metabolic panel) | 1 | 0 | 0 |
| Medical History | Lifestyle | 1 | 0 | 0 |
| Medical History | Medical history (intraoperative details from organ recovery surgery e.g. blood pressure or ventilator settings) | 1 | 0 | 0 |
| Medical History | Medical history (publish only with consent of donor's family/legal representative) | 1 | 0 | 0 |
| Medical History | Past infections | 1 | 0 | 0 |
| Medical History | Pediatric medical history - history of genetic anomalies | 1 | 0 | 0 |
| Medical History | Pediatric medical history - history of preterm birth | 1 | 0 | 0 |
| Medical History | Pediatric medical history - hypoplastic organs | 1 | 0 | 0 |
| Medical History | Physiological measurements (body measurements (not specified)) (publish only with consent of donor's family/legal representative) | 1 | 0 | 0 |
| Medical History | Physiological measurements (oxygen saturations) | 0 | 1 | 0 |
| Medical History | Previous surgeries | 1 | 0 | 0 |
| Medical History | Substance use (alcohol) | 1 | 0 | 0 |
| Medical History | Substance use (cannabis smoking) | 1 | 0 | 0 |
| Medical History | Substance use (drug use) | 1 | 0 | 0 |
| Demographic Information | Race | 11 | 0 | 0 |
| Demographic Information | Age | 10 | 0 | 0 |
| Demographic Information | Ethnicity | 9 | 0 | 0 |
| Demographic Information | Sex | 8 | 0 | 0 |
| Demographic Information | Gender | 6 | 0 | 0 |
| Demographic Information | Age (publish only with consent of donor's family/legal representative) | 1 | 0 | 0 |
| Demographic Information | Demographic Information (general or unspecified) | 1 | 0 | 0 |
| Demographic Information | Race (participant emphasized race should only be reported with donor consent) | 0 | 1 | 0 |
| Demographic Information | Race (publish only with consent of donor's family/legal representative) | 1 | 0 | 0 |
| Personally Identifying Information | Personally identifying information (general or unspecified) | 0 | 1 | 3 |
| Personally Identifying Information | Zip code | 0 | 1 | 2 |
| Personally Identifying Information | Address | 0 | 0 | 2 |
| Personally Identifying Information | Name | 0 | 0 | 2 |
| Personally Identifying Information | HIPAA-protected information (any) | 0 | 0 | 1 |
| Personally Identifying Information | Other personally identifying information aside from address and telephone number | 0 | 1 | 0 |
| Personally Identifying Information | Social Security Number | 0 | 1 | 0 |
| Personally Identifying Information | Socioeconomic disadvantage score (based on zip code) | 1 | 0 | 0 |
| Personally Identifying Information | Telephone number | 0 | 0 | 1 |
| Personally Identifying Information | UNOS ID | 0 | 0 | 1 |
| Sociodemographic Information | Socioeconomic status | 4 | 1 | 1 |
| Sociodemographic Information | Location (broad geographic region of donor residence) | 2 | 2 | 1 |
| Sociodemographic Information | Education level | 1 | 0 | 0 |
| Sociodemographic Information | Social history | 1 | 0 | 0 |
| Tissue or Organ Sample Features | Anatomical region sample was taken from | 1 | 0 | 0 |
| Tissue or Organ Sample Features | Fitzpatrick scale (skin) | 1 | 0 | 0 |
| Tissue or Organ Sample Features | Molecular and genomic data (RNA sequence data) | 0 | 1 | 0 |
| Tissue or Organ Sample Features | Molecular and genomic data (epigenetic data) | 0 | 1 | 0 |
| Tissue or Organ Sample Features | Molecular and genomic data (genomic/sequencing data) | 0 | 1 | 0 |
| Tissue or Organ Sample Features | Proximity to tumor for non-tumorous samples removed from a cancerous organ | 1 | 0 | 0 |
| Tissue or Organ Sample Features | Unusual substances/unknown species detected in the sample | 1 | 0 | 0 |
| Cause of Death | Cause of death | 3 | 1 | 0 |
| Cause of Death | Mechanism of death | 2 | 1 | 0 |
| Tissue or Organ Sample Quality and Viability | Cold ischemic time | 1 | 0 | 0 |
| Tissue or Organ Sample Quality and Viability | Processing time | 1 | 0 | 0 |
| Tissue or Organ Sample Quality and Viability | Warm ischemic time | 1 | 0 | 0 |
