# Supplementary material for: Researchers’ perspectives on donor-level metadata collection and reporting for human tissue research
Source: JAMIA Open. 2026 Nov 6;9(6):ooag213. doi: 10.1093/jamiaopen/ooag213 (PMC13743425; doi:10.1093/jamiaopen/ooag213)
Supplement: Supplementary file 1 [file ooag213_supplementary_data.zip › JAMIA Appendix F.docx]

Appendix F. Illustrative crosswalk of participant-informed donor-level metadata recommendations with existing biomedical informatics frameworks and terminologies.

| **Participant-informed metadata category** | **Metadata element** | **Illustrative representation within existing biomedical informatics frameworks and terminologies** |
| --- | --- | --- |
| **Demographic information** | Age | FHIR Patient; OMOP Person; MIABIS Sample Donor |
|  | Race | FHIR US Core Patient (Race Extension); OMOP Person |
|  | Ethnicity | FHIR US Core Patient (Ethnicity Extension); OMOP Person |
|  | Sex | FHIR US Core Patient (Individual Sex Extension); OMOP Person; MIABIS Sample Donor |
|  | Gender | FHIR Patient (Individual Gender Identity Extension); OMOP Observation |
| **Medical history** | Physiological measurements | FHIR Observation; OMOP Measurement; LOINC |
|  | Laboratory test results | FHIR Observation; OMOP Measurement; LOINC |
|  | Diagnosed medical conditions | FHIR Condition; OMOP Condition Occurrence; SNOMED CT |
|  | Medical history (general) | FHIR Condition or Observation; OMOP Observation; SNOMED CT, where applicable |
|  | Substance use | FHIR Observation or QuestionnaireResponse; OMOP Observation; SNOMED CT where applicable |
|  | Medication history | FHIR MedicationStatement; OMOP Drug Exposure; RxNorm |
|  | Dietary history | FHIR Observation or QuestionnaireResponse; OMOP Observation |
|  | Pregnancy-related medical history | FHIR Condition or Observation; OMOP Condition Occurrence or Observation or Measurement; SNOMED CT or LOINC, depending on the metadata element |
|  | Embryo/fetus/infant medical history | FHIR Condition or Observation; OMOP Condition Occurrence or Observation or Measurement; SNOMED CT or LOINC, depending on the metadata element |
|  | Pediatric medical history | FHIR Condition or Observation; OMOP Condition Occurrence or Observation or Measurement; SNOMED CT or LOINC, depending on the metadata element |
| **Cause of death** | Cause of death | FHIR Observation; OMOP Death; SNOMED CT, where applicable |
|  | Mechanism of death | FHIR Observation; OMOP Observation; SNOMED CT, where applicable |
| **Sociodemographic information** | Sociodemographic context | FHIR Observation or QuestionnaireResponse; OMOP Observation |
| **Personally identifying information** | Personally identifying information | FHIR Patient; OMOP Person or Location |

Abbreviations: FHIR, Fast Healthcare Interoperability Resources; OMOP, Observational Medical Outcomes Partnership; MIABIS, Minimum Information About BIobank data Sharing; SNOMED CT, Systematized Nomenclature of Medicine Clinical Terms; LOINC, Logical Observation Identifiers Names and Codes; RxNorm, normalized clinical drug terminology.

FHIR, OMOP, and MIABIS provide data structures for representing metadata, while SNOMED CT, LOINC, and RxNorm provide standardized clinical terminologies that may support coding of specific metadata elements.

Note: This crosswalk is illustrative rather than prescriptive. Mappings identify existing biomedical informatics frameworks and terminologies that may support representation of the participant-informed donor-level metadata recommendations. The mappings do not constitute a validated implementation specification or proposed metadata standard, and specific mappings may vary depending on how individual metadata elements are defined and operationalized, as well as across framework versions and implementations.
