# Supplementary material for: Researchers’ perspectives on donor-level metadata collection and reporting for human tissue research
Source: JAMIA Open. 2026 Nov 6;9(6):ooag213. doi: 10.1093/jamiaopen/ooag213 (PMC13743425; doi:10.1093/jamiaopen/ooag213)
Supplement: Supplementary file 1 [file ooag213_supplementary_data.zip › JAMIA Appendix G.docx]

## **Appendix F. CReDit Author Statement**

| RLG | Conceptualization; Formal analysis; Methodology; Visualization; Writing – original draft; Writing – review & editing |
| --- | --- |
| KT | Data curation; Formal analysis; Methodology; Validation; Writing – review & editing |
| MK | Conceptualization; Data curation; Investigation; Project administration; Validation; Writing – original draft; Writing – review & editing |
| ME | Writing – review & editing; Validation |
| PS | Funding acquisition; Writing – review & editing |
| YLS | Conceptualization; Funding acquisition; Methodology; Project administration; Resources; Supervision; Writing – original draft; Writing – review & editing |
